# Treg derived Amphiregulin protects from murine lupus nephritis via tissue reparative effects

**DOI:** 10.1038/s41598-025-91636-2

**Published:** 2025-03-05

**Authors:** Laura-Isabell Ehnold, Simon Melderis, Julia Hagenstein, Matthias T. Warkotsch, Viona Laas, Frederic C. Feindt, Hui Wu, Tobias B. Huber, Florian Grahammer, Oliver M. Steinmetz

**Affiliations:** 1 III. Department of Medicine, University Medical Center Hamburg-Eppendorf, Martinistrasse 52, 20246 Hamburg, Germany; 2 Hamburg Center for Kidney Health, University Medical Center Hamburg-Eppendorf, Hamburg, Germany

**Keywords:** Lupus nephritis, Cytokines

## Abstract

Systemic lupus erythematosus (SLE) is a common autoimmune disease that affects multiple organ systems. Among the most severe manifestations of SLE is lupus nephritis (LN), which causes particularly high morbidity. Recently, we identified amphiregulin (AREG), an epidermal growth factor receptor ligand, as a key mediator of LN via downregulation of pathogenic CD4^+^ T-cell responses. In human LN, AREG is mainly produced by regulatory T cells (Tregs) and monocytes/macrophages (M/M). Since AREG´s functions have been shown to vary considerably depending on the source, we aimed to clarify the cell-type-specific roles of AREG using the pristane model of LN. Conditional knockout mice lacking Treg- but not M/M-derived AREG showed worse LN outcome at 12 and 15 months with increased glomerular cell proliferation, apoptosis and renal tissue fibrosis. Interestingly, immune responses were not relevantly affected by the lack of AREG from either leukocyte source, indicating a different mechanism. In this respect, in vitro studies demonstrated improved wound healing of murine mesangium and tubulus cells and enhanced regeneration and sprouting of human glomerular endothelial cells after incubation with recombinant AREG. These findings underscore the importance of Treg-derived AREG in tissue regeneration and protection from fibrosis in LN, highlighting AREG as a potential therapeutic target.

**Supplementary Information:**

The online version contains supplementary material available at 10.1038/s41598-025-91636-2.

## Introduction

Systemic lupus erythematosus (SLE) is a common autoimmune disease, known to cause high morbidity and mortality among younger patients^1^. Renal involvement, termed Lupus nephritis (LN), significantly worsens prognosis^2^. Despite its high prevalence, the underlying causes and mechanisms of LN remain poorly understood^3^. Currently, available therapeutic options are nonspecific, cause relevant side effects, and often fail to induce remission of disease. This causes high patient morbidity, including end-stage renal disease at a young age^4,5^. Consequently, there is an urgent need for the development of novel and targeted therapeutic approaches. Amphiregulin (AREG), a multifunctional cytokine, that binds to the epidermal growth factor receptor (EGFR), has emerged as a potential player in the pathogenesis of SLE and has been shown to be significantly upregulated in peripheral blood leukocytes of SLE patients^6^. AREG is known to influence various biological processes, including tissue repair, cellular differentiation, and immune regulation^7–9^. In the context of SLE, our group has recently shown a protective role of AREG in the model of pristane oil-induced LN via broad downregulation of CD4^+^ T cell activation by direct interaction^10^. It is noteworthy, that agents targeting EGFR signaling are already utilized in cancer treatment, indicating AREG’s clinical relevance. However, AREG’s functions are complex, exhibiting tissue protective anti-inflammatory and repair functions but also pro-inflammatory and pro-fibrotic properties. These differential functions seem to depend largely on the cellular source of AREG secretion. Macrophage-derived AREG, for example, demonstrated a protective role in both, a model of uveitis^11^ and experimental cardiac injury^12^. A landmark study by Minutti et al. could show, that AREG derived from macrophages initiates the activation of TGF-β, which in turn supports the differentiation of pericytes into myofibroblasts and thus supports effective vascular healing^13^. In addition to macrophages, regulatory T cells (Tregs) also express AREG. Surprisingly, Treg-derived AREG does not have immunosuppressive effects but rather supports tissue repair as shown in models of muscle and lung injury^14,15^. Furthermore, recent studies have demonstrated, that AREG secreted by different innate cells, such as macrophages, mast cells and basophils, strongly enhances the immunosuppressive functions of Tregs, via mechanisms that remain elusive^11^, 16]–^18^.

Contrary to these anti-inflammatory and tissue repair functions, multiple studies have, however, also reported a dark side of AREG in inflammation. AREG exhibits pro-inflammatory functions in various autoimmune conditions, such as psoriasis, rheumatoid arthritis, Sjögren´s syndrome and allergic asthma. These Studies have demonstrated, that AREG plays a role in the production of pro-inflammatory cytokines within tissues, which exacerbates the underlying diseases^19–22^. Furthermore, our group has previously demonstrated, that AREG secreted by resident renal cells induces and stimulates pro-inflammatory M1 type macrophages, thereby exacerbating renal injury in a model of acute glomerulonephritis^23^. In addition to its pro-inflammatory effects, AREG can also contribute to increased fibrosis, as shown in models of liver and lung injury^24,25^. The influence of AREG on fibrosis has also been demonstrated in the kidney. Renal AREG expression was shown to correlate with the severity of fibrosis in both acute and chronic renal diseases^26^. Furthermore, selective knockout of AREG in proximal tubule cells conferred protection against fibrosis in mouse models of unilateral ureteral obstruction and ischemia-reperfusion injury^27^. In summary, AREG fulfills multiple and partly contradictory roles, which are strongly context-dependent and vary according to the cellular source. AREG derived from resident tissue cells seems to be pro-inflammatory and pro-fibrotic, whereas AREG derived from leukocytes is predominantly anti-inflammatory and tissue-reparative. This concept was recently challenged by a study showing, that Treg-derived AREG, which had previously been shown to be tissue reparative, can also enhance detrimental liver fibrosis in the model of non-alcoholic steatosis^28^. The multiple and cell type-specific differential roles of AREG secretion thus remain poorly understood. Given our previous observation, that AREG protects from LN, we aimed to follow up on this notion. Since our data had revealed Tregs and macrophages as the strongest renal AREG producers in patients with LN^10^, we set off to analyze the cell type-specific roles of AREG derived from these cells, to identify novel AREG/EGFR-based therapeutic options.

## Results

### Deficiency of AREG from Tregs leads to a worsened renal outcome in Pristane-induced LN

**Fig. 1:**
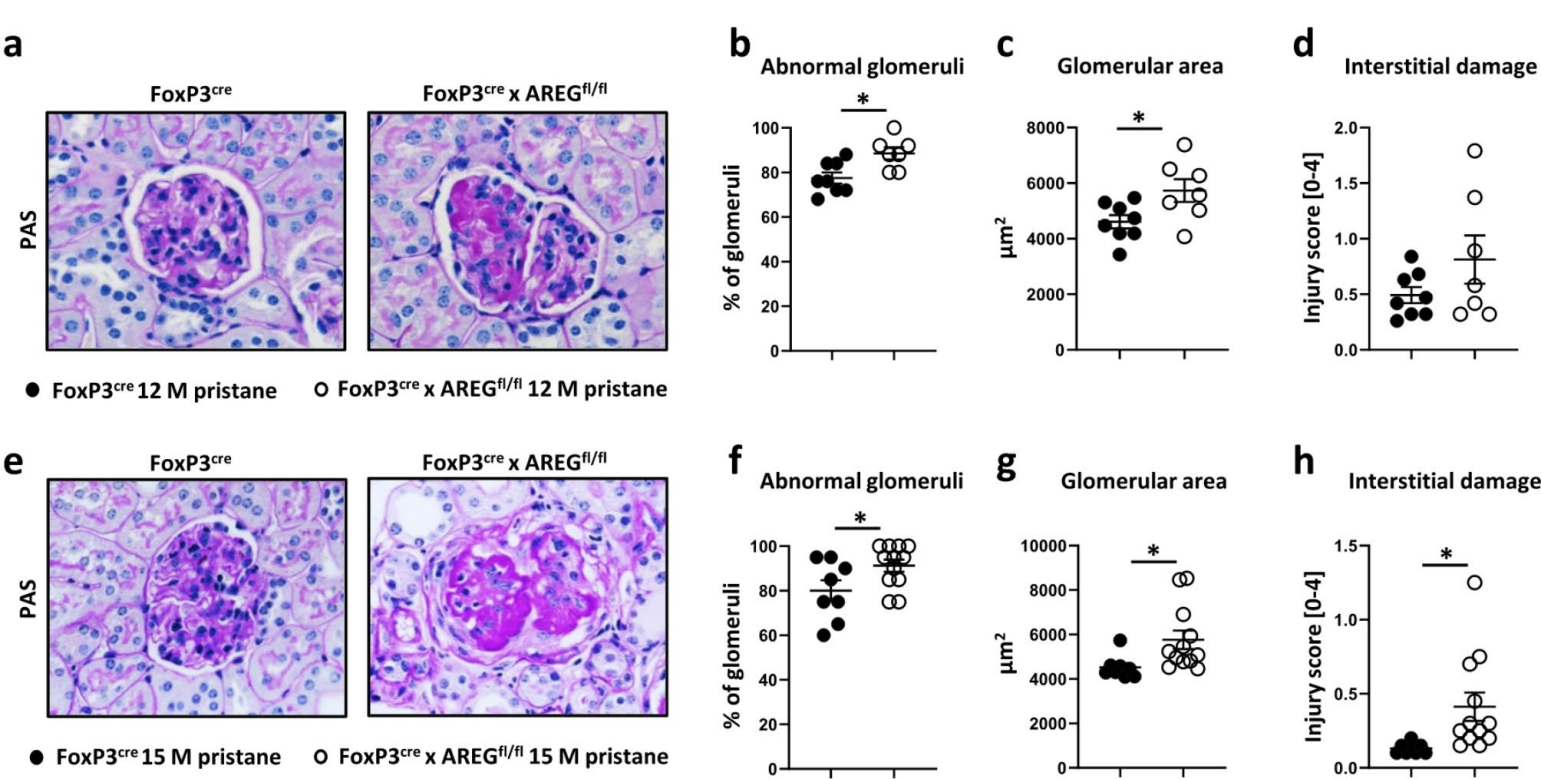
Deficiency of Treg derived AREG aggravates LN. (**a**) Representative PAS-stained kidney sections from the indicated mouse strains at 12 months of pristane-induced LN. (**b**-**d**) Quantification of (**b**) abnormal glomeruli, (**c**) glomerular area and (**d**) interstitial injury at 12 months after LN induction. (**e**) Representative PAS-stained sections of kidneys from the indicated strains of mice at 15 months of pristane-induced LN. (**f**-**h**) Quantification of (**f**) abnormal glomeruli, (**g**) glomerular area and (h) interstitial injury at 15 months after LN induction. Circles show individual animals, horizontal lines show mean values. Error bars show the standard error of the mean. *p < 0.05.

To investigate the role of Treg-derived AREG in LN, we utilized cre-flox mice with targeted AREG depletion under the control of the FoxP3 promoter, referred to as Foxp3^cre^ x AREG^fl/fl^ mice. Effective AREG excision in Tregs was validated by PCR analysis of targeted critical exons 3 and 4 from genomic DNA of FACsorted B-cells, Teffs, and Tregs, as well as tail snips of Foxp3^cre^ x AREG^fl/fl^ mice (Sup. Fig S1a-c). For protein level validation, we induced LN by intraperitoneal pristane oil injection. FACS analysis of spleen and kidney cells indeed demonstrated almost absent AREG expression in Tregs from pristane-treated Foxp3^cre^ x AREG^fl/fl^ mice (Sup. Fig. S1d-f), while expression by γδ T cells remained unchanged (Sup. Fig. S1g-i). After 12 months of pristane-induced LN, we examined kidney histology, which revealed a significant increase in abnormal glomeruli (Fig. 1a, b) and glomerular area in mice lacking AREG in Tregs (Fig. 1c). At this time point, the renal interstitium only showed a trend towards more damage (Fig. 1d). Continuing our investigation, we sought to explore even longer-term effects by extending the disease period to 15 months. Again, mice lacking AREG expression in Tregs exhibited a significant increase in abnormal glomeruli and showed an enlarged glomerular area (Fig. 1e, f, g). Additionally, the interstitial damage was notably more severe at this later time point (Fig. 1h). As a control, we also compared glomerular and interstitial injury, as well as the glomerular size of naïve, aged wild type and Foxp3^cre^ x AREG^fl/fl^ mice. Different from disease, in the naïve situation, Foxp3^cre^ x AREG^fl/fl^ mice showed no relevant renal injury and even slightly smaller glomeruli than their AREG-sufficient counterparts (Sup. Fig. S2). The observed reno-protective role of Treg-derived AREG thus indeed depends on the chronic inflammatory context of LN. In sum, these results suggest an enduring protective role of AREG derived from Tregs in mitigating renal histological damage in pristane-induced LN.

### Treg-derived AREG ameliorates renal fibrosis

**Fig. 2:**
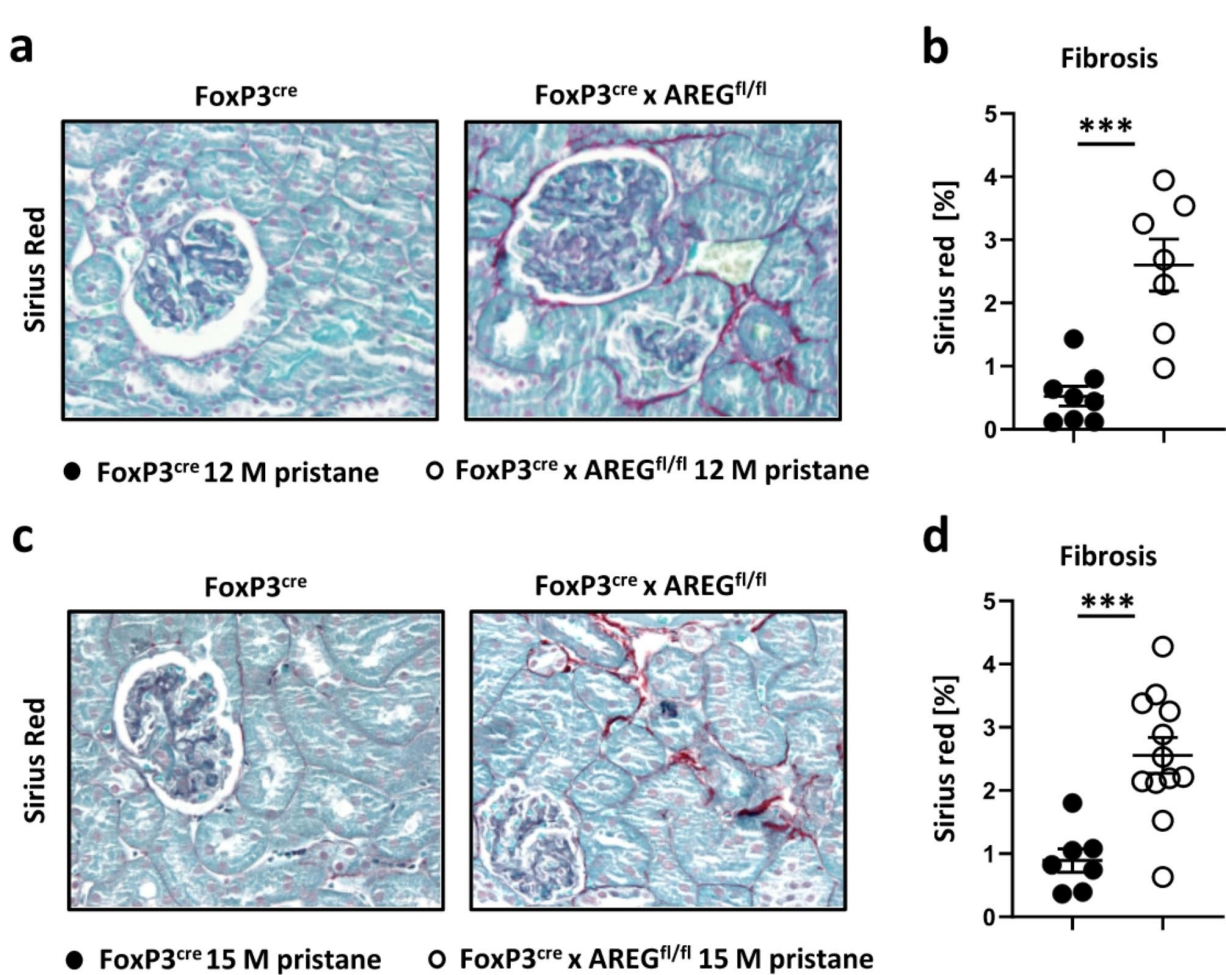
Treg derived AREG ameliorates renal fibrosis. (**a**) Representative Sirius Red stained sections of kidneys from FoxP3^cre^ controls and FoxP3^cre^ x AREG^fl/fl^ mice at 12 months of pristane-induced LN. (**b**) Quantification of fibrosis at 12 months after LN induction (**c**) Representative Sirius Red stained sections of kidneys from FoxP3^cre^ and FoxP3^cre^ x AREG^fl/fl^ mice at 15 months of pristane-induced LN. (**d**) Quantification of fibrosis at 15 months after LN induction. Circles show individual animals and horizontal lines show mean values. Error bars show the standard error of the mean. *** *p* < 0.001.

Recent insights have highlighted the crucial role of AREG in orchestrating the balance between tissue repair and fibrosis. We therefore aimed to study this aspect in our model of LN. Remarkably, the absence of Treg-derived AREG resulted in a significant increase of fibrosis at 12 months of pristane-induced LN (Fig. 2a, b). Notably, age-matched naïve mice with AREG deficient Tregs displayed no elevated fibrosis, underscoring the specificity of the observed effect in the context of chronic renal inflammation (Sup. Fig. S3). The increase in fibrosis persisted even at 15 months and was consistently higher than in the control group. (Fig. 2c, d).

### The absence of Treg-derived AREG does not exacerbate cellular immune responses

**Fig. 3:**
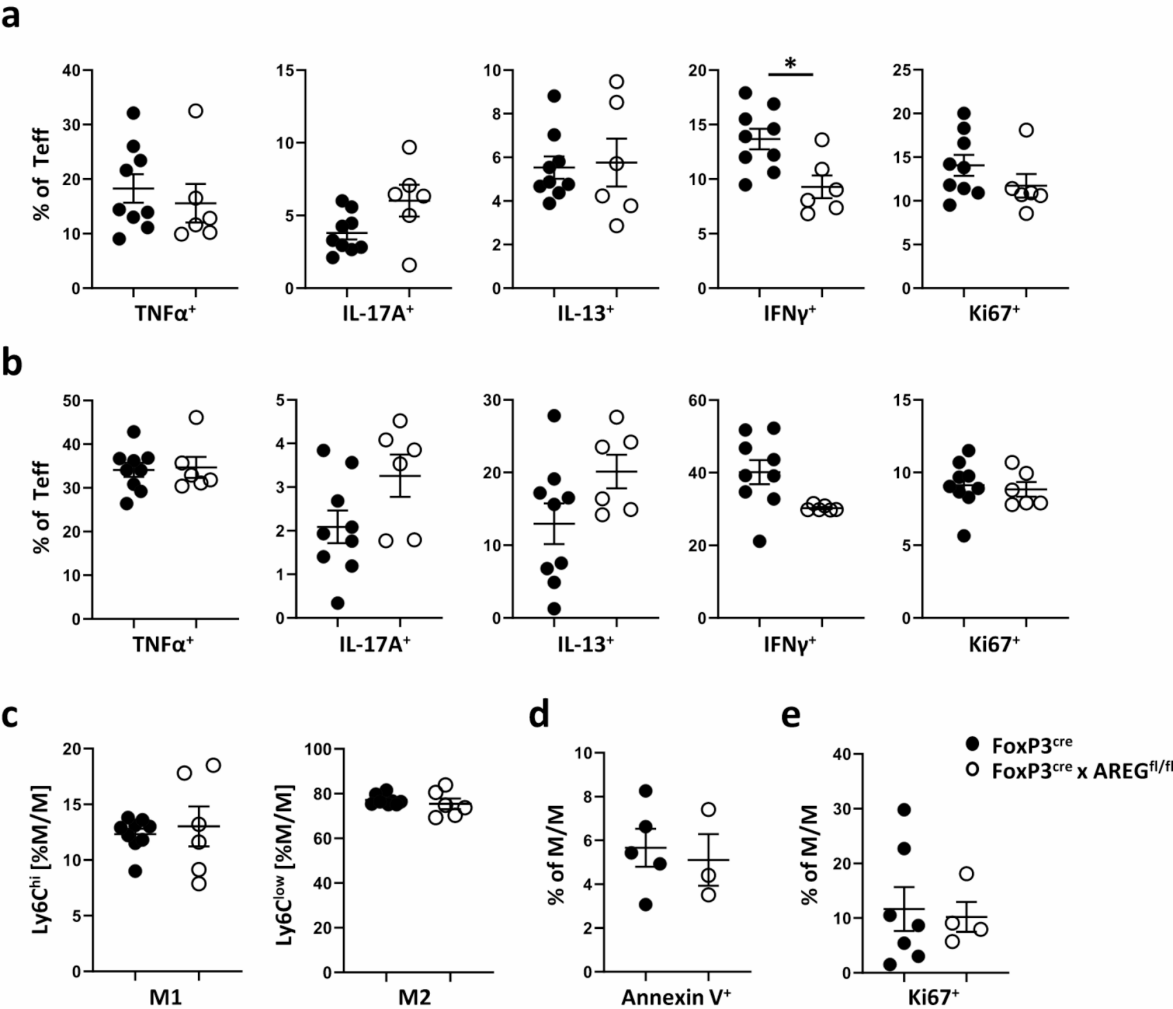
Lack of AREG from Tregs does not exacerbate cellular immune responses. (**a**) Cytokine production and proliferation (Ki67) of Teffs in kidneys from mice of the indicated strains at 12 months of pristane-induced LN. (**b**) Cytokine production and proliferation of Teffs from spleens of the indicated strains of mice at 12 months of pristane-induced LN. (**c**) FACS analysis of kidney monocyte/macrophage (M/M) polarization into pro- (M1) and anti-inflammatory (M2) macrophages as assessed by expression of Ly6C in the indicated strains of mice after 12 months of pristane-induced LN. (**d**) M/M apoptosis, as measured by annexin V staining of pristane-induced peritoneal macrophages in the indicated strains of mice. (**e**) Proliferation of macrophages as measured by Ki67 staining of pristane-induced peritoneal macrophages in the indicated strains of mice. Circles show individual animals, horizontal lines show mean values. Error bars show the standard error of the mean. *p < 0.05.

An earlier study from our lab had demonstrated a broad anti-inflammatory effect of AREG on T effector cell responses^10^. Therefore, considering the observation of increased fibrosis in FoxP3^cre^ x AREG^fl/fl^ mice, we hypothesized that this could be attributed to increased nephritogenic immune responses in the absence of Treg-derived AREG. However, T effector cells inside the kidneys of nephritic FoxP3^cre^ x AREG^fl/fl^ mice did not exhibit increased expression of pro-inflammatory cytokines. On the contrary, IFNγ secretion was even slightly reduced at 12 but not 15 months after induction of LN. Moreover, renal Teff proliferation remained unaffected (Fig. 3a, Sup. Fig. S4a). Similarly, Teff in the spleen did not demonstrate significant differences in their cytokine production or proliferation at either time point (Fig. 3b, Sup. Fig. S4b). The gating strategy used for renal and splenic Teff is shown in Supplementary Figure S5. In another previously published study, we had also demonstrated, that AREG derived from renal resident cells promotes the polarization of pro-inflammatory M1 macrophages and protects them from apoptosis in a mouse model of acute glomerulonephritis^23^. However, macrophages present in the kidneys of FoxP3^cre^ x AREG^fl/fl^ mice at 12 months after pristane nephritis induction displayed no significant differences in their M1/M2 polarization (Fig. 3c). The gating strategy is shown in Supplementary Figure S6. To also assess the possible influence of Treg-derived AREG on M/M apoptosis, we analyzed macrophages activated by pristane-induced peritonitis. In contrast to the situation in AREG pan-knockout mice, we did not observe any noticeable differences regarding apoptosis (Fig. 3d) or proliferation (Fig. 3e), when absence of AREG was restricted to Tregs. In summary, the results described above indicate, that Treg-derived AREG lacks significant effects on activation and cytokine secretion of CD4^+^ Teff or macrophages. This suggests, that an alternative mechanism underlies the observed renoprotection in LN mediated by Treg-derived AREG.

### Lack of AREG from Tregs does not affect humoral immune responses

**Fig. 4:**
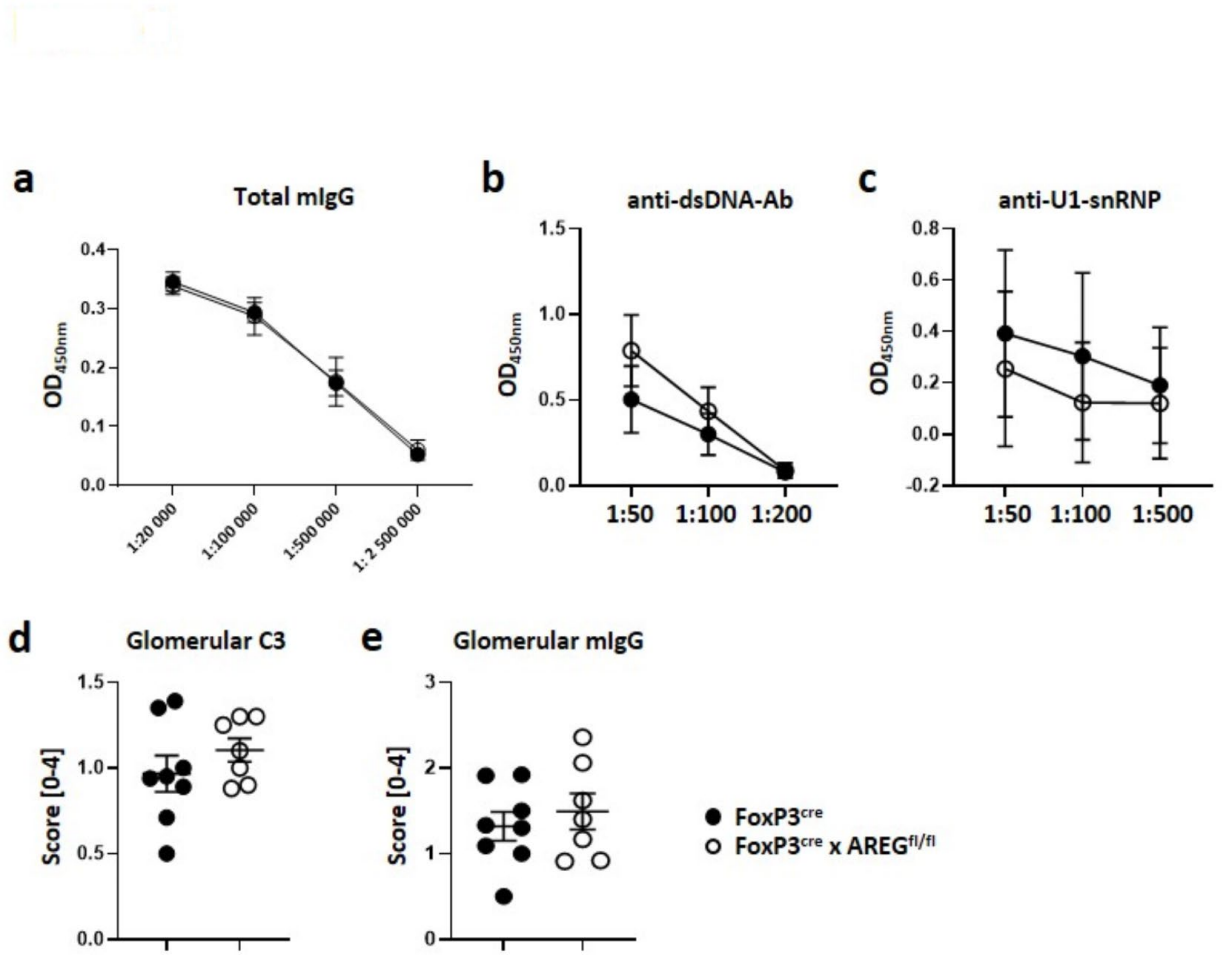
Lack of AREG from Tregs does not affect humoral immune responses. (**a**-**c**) Analysis of serum (**a**) total mouse IgG, (**b**) anti-dsDNA antibodies and (**d**) anti-U1-snRNP antibodies at 12 months of pristane-induced LN in the indicated strains of mice at the indicated dilutions. (**d**-**e**) Quantification of (**d**) C3 and (**e**) mouse IgG deposition in glomeruli at 12 months of pristane-induced LN in the indicated strains of mice. OD: optical density. Circles in a-c show mean values and bars show the standard error of the mean. Circles in (**d**) + (**e**) show individual animals and horizontal lines show mean values. Error bars show the standard error of the mean.

Next, we turned our attention to examining SLE characteristic humoral immune responses. However, at 12 months of pristane-induced LN, mice lacking AREG from Tregs did not show significant differences in serum total IgG levels (Fig. 4a). Even more importantly, the prototypical SLE markers, anti-dsDNA antibodies (Fig. 4b) and anti-U1-snRNP antibodies (Fig. 4c), also remained unaffected in the absence of Treg-derived AREG. Given our group’s recent demonstration, that the complete absence of AREG results in increased glomerular C3 and IgG deposition in LN^10^, we also explored this aspect in Foxp3^Cre^ x AREG^fl/fl^ mice. However, no change in glomerular C3 (Fig. 4d) or mouse IgG (Fig. 4e) deposition was observed. Taken together, our data thus far make it unlikely, that Treg-derived AREG mediates protective effects in LN through immunomodulation.

### M/M-derived AREG does not relevantly alter the course of pristane-induced LN

**Fig. 5:**
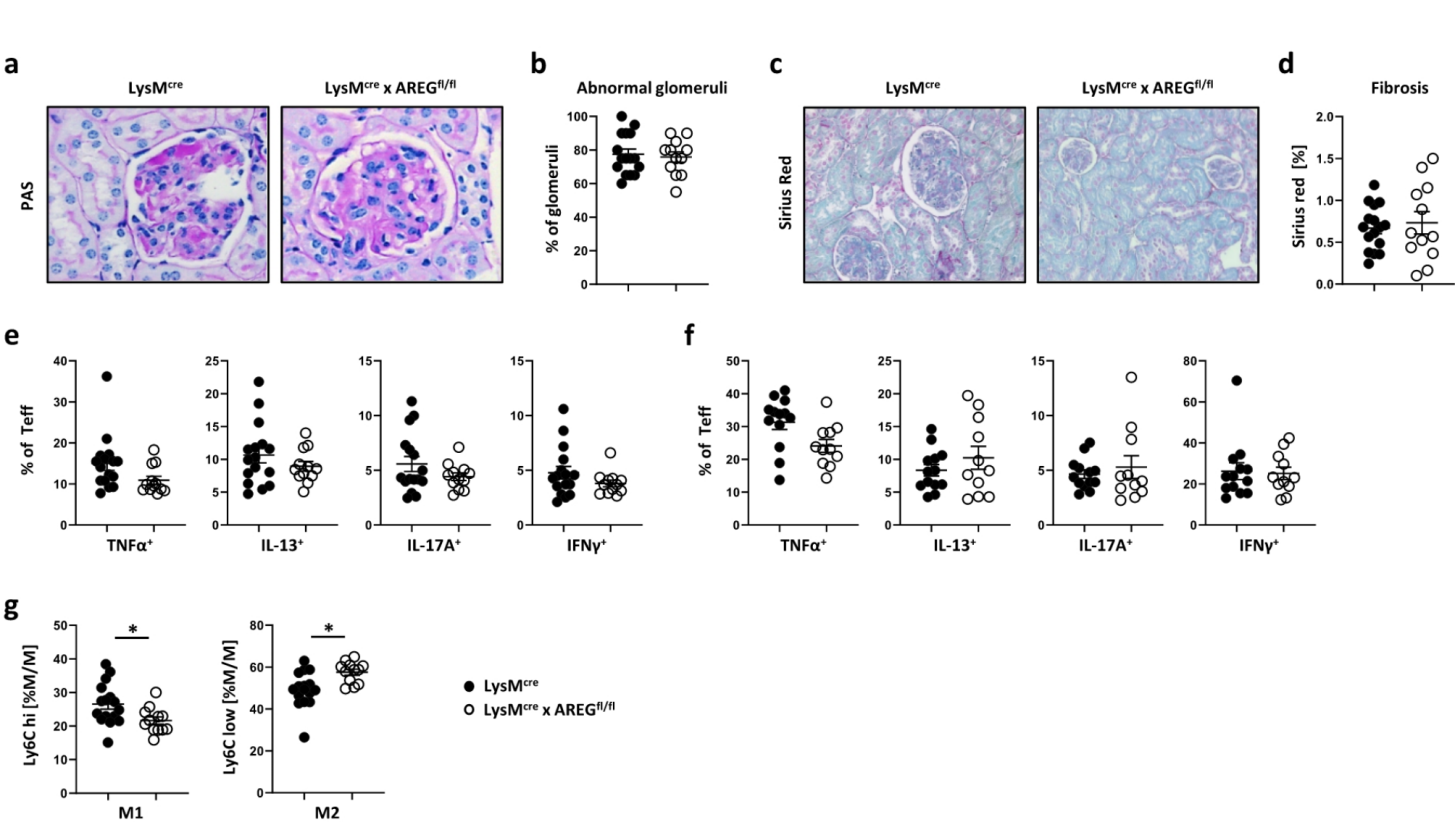
M/M-derived AREG does not alter renal outcome or fibrosis in LN. (**a**) Representative PAS-stained sections of kidneys from the indicated strains of mice at 12 months of pristane-induced-LN. (**b**) Quantification of abnormal glomeruli in kidneys of LysM^cre^ x AREG^fl/fl^ and control mice at 12 months of pristane-induced LN. (**c**) Representative Sirius Red-stained sections of kidneys from the indicated strains of mice at 12 months of pristane-induced LN. (**d**) Quantification of fibrosis in kidneys of LysM^cre^ x AREG^fl/fl^ and control mice at 12 months of pristane-induced LN. (**e**-**f**) Cytokine expression by Teffs in the (**e**) kidney and spleen (**f**) of LysM^cre^ x AREG^fl/fl^ mice or controls at 12 months of pristane-induced LN. (**g**) Kidney M/M polarization into pro- (M1) and anti-inflammatory (M2) phenotypes as measured by expression of Ly6C in the indicated strains of mice at 12 months after pristane injection. Symbols show individual animals and horizontal lines show mean values. Error bars show the standard error of the mean. *p < 0.05.

In addition to AREG produced by Tregs, M/M-secreted AREG is recognized for its potent tissue-protective effects^13^. Furthermore, our previous studies in human LN identified M/M as a significant source of renal AREG expression^10^. Similar to the situation in humans, M/M constituted a relevant fraction of renal AREG producing immune cells, which was significantly expanded during pristane induced LN (Sup. Fig. S7). Therefore, to explore this notion further, we generated LysM^cre^ x AREG^fl/fl^ mice (Sup. Fig. S8), eliminating AREG production in most myeloid cell lineages, including monocytes and macrophages^13^. Renal damage assessment at 12 months after induction of LN, however, revealed no significant differences between mice lacking AREG in myeloid cells and control mice (Fig. 5a, b). Similarly, examination of renal fibrosis showed no significant distinctions between the two groups (Fig. 5c, d). Investigating potential immunological phenotypes, linked to loss of AREG from myeloid cells, we found that cytokine production from Teffs exhibited no significant differences in kidneys (Fig. 5e) or spleens (Fig. 5**f**), recapitulating our observations in mice with Treg-restricted AREG deficiency. Interestingly, as a secondary finding, we noted a slight but significant decrease in M1 and an increase in M2 polarization in renal macrophages lacking AREG production (Fig. 5**g**). Collectively, our findings indicate, that the tissue-protective effects of AREG in LN are specifically mediated by Treg- but not M/M-derived AREG. Conversely, myeloid cell-restricted AREG deficiency predominantly promotes polarization towards anti-inflammatory M2 macrophages with, however, no discernible impact on fibrosis and the overall renal outcome in LN. Consequently, we aimed to delve into the hitherto unclear mechanisms underlying the observed renoprotective effects of Treg-derived AREG.

#### Treg-derived AREG protects resident renal cells from apoptosis and enhances tubular repair

**Fig. 6:**
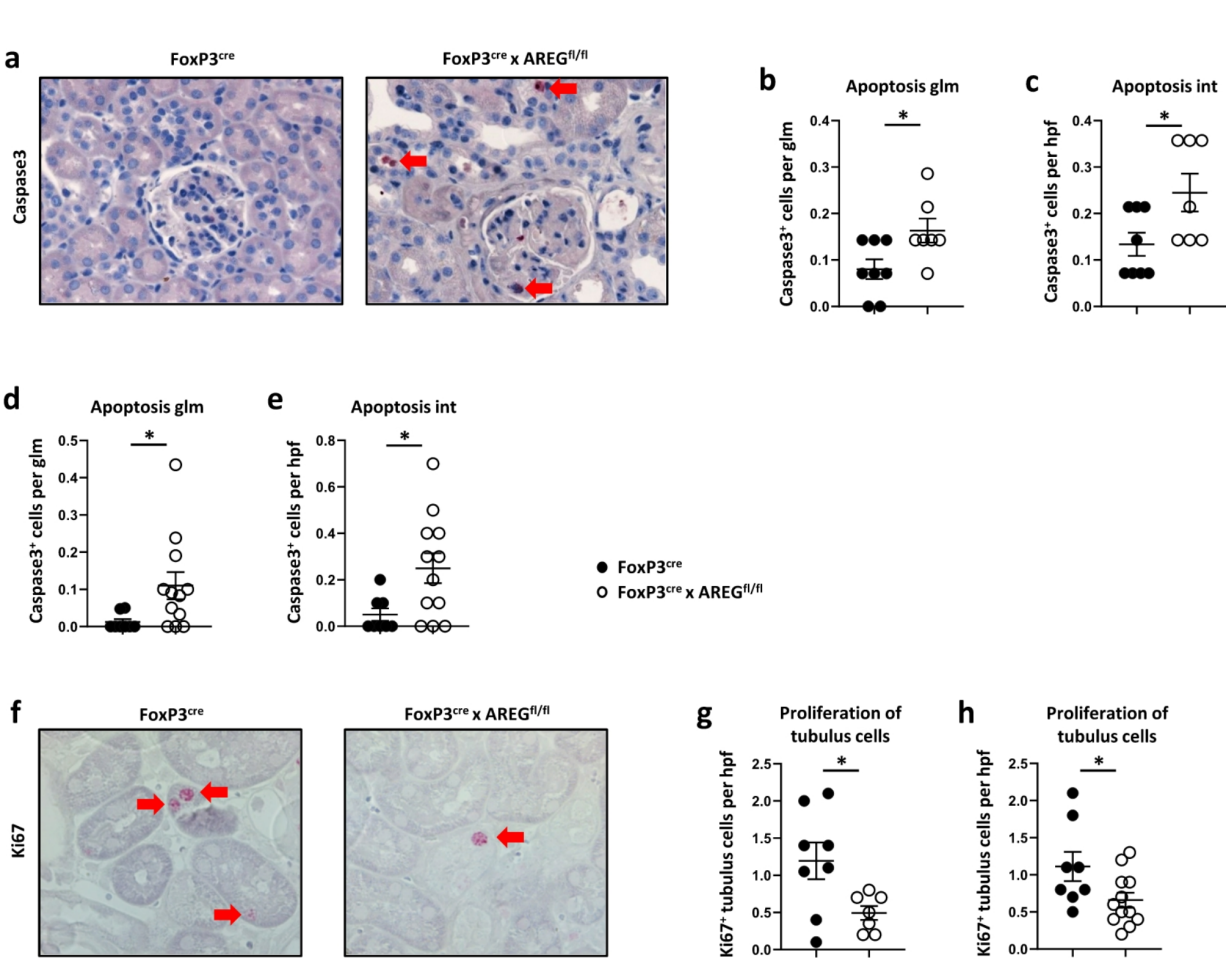
Treg-derived AREG protects renal cells from apoptosis and enhances tubular repair. (**a**) Representative photographs of caspase 3 stained (red) kidney sections from FoxP3^cre^ controls and FoxP3^cre^ x AREG^fl/fl^ mice at 12 months of pristane-induced LN. Arrows indicate positive cells. (**b**) Quantification of caspase 3 positive cells per glomerulus (glm) at 12 months after LN induction. (**c**) Caspase 3 positive cells per high power field (hpf) in the renal interstitium (int) at 12 months after LN induction. (**d**) Quantification of caspase 3 positive cells per glomerulus (glm) at 15 months after LN induction. (**e**) Quantification of caspase 3 positive cells per high power field (hpf) in the interstitium at 15 months after LN induction. (**f**) Representative photographs of Ki67 stained (red) kidney sections from FoxP3^cre^ controls and FoxP3^cre^ x AREG^fl/fl^ mice at 12 months of pristane-induced LN. Arrows indicate positive cells. (**g**) Quantification of Ki67 positive tubulus cells per high power field (hpf) at 12 months after LN induction. (**h**) Quantification of Ki67 positive tubulus cells per high power field (hpf) at 15 months after LN induction. Circles show individual animals and horizontal lines show mean values. Error bars show the standard error of the mean. *p < 0.05.

LN is known to increase apoptosis of resident renal cells. This is also the case in the pristane-induced LN model, as shown in Supplementary Figure S9. We thus studied the impact of Treg-derived AREG on cleaved caspase 3-expressing apoptotic cells within renal tissue. At 12 months of pristane-induced LN, kidney sections from FoxP3^cre^ x AREG^fl/fl^ mice indeed exhibited increased counts of apoptotic cells in the glomeruli, as well as in the interstitium (Fig. 6a-c). The increase in the number of cleaved caspase 3-expressing cells persisted even after 15 months and was consistently higher in both glomeruli and interstitium of mice lacking AREG from Tregs than in the control group (Fig. 6d, e). Next, we aimed to assess, whether AREG would also enhance repair of tubular epithelial cells and thus studied expression of the proliferation marker Ki67. Indeed, immunohistochemical analyses showed a significant reduction of proliferating tubulus cells in FoxP3^cre^ x AREG^fl/fl^ mice at both 12 and 15 months, indicating a relevant role of AREG for regeneration of injured renal tissue (Fig. 6f-h).

### Recombinant AREG improves wound healing and enhances angiogenesis

**Fig. 7:**
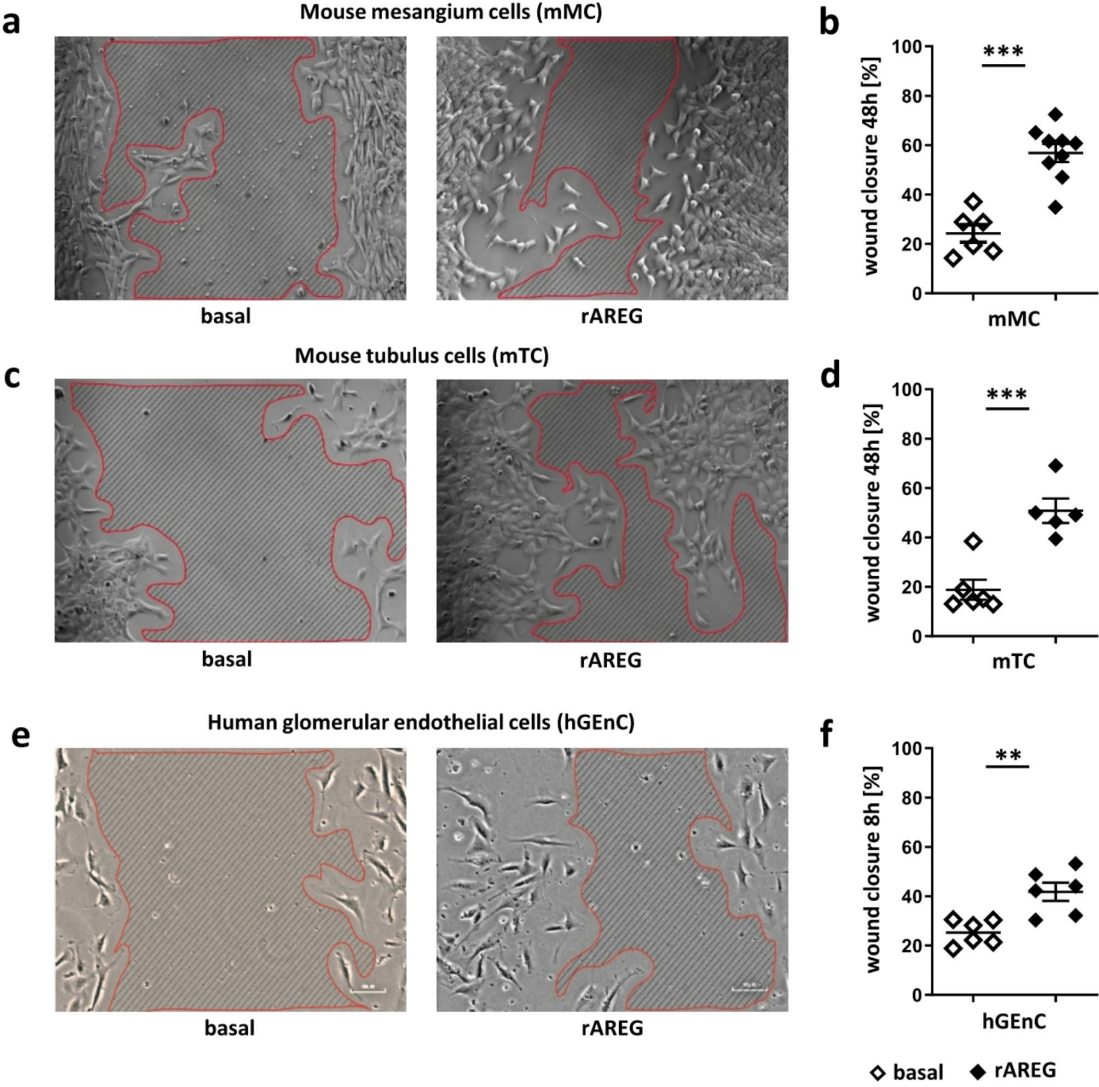
Recombinant AREG leads to improved wound healing. (**a**) Representative photographs of wound closure at 48 h after application of a scratch to a layer of murine mesangium cells (mMC) with or without recombinant AREG (rAREG). The remaining wound area is hatched and circled by the red line. (**b**) Quantification of wound closure at 48 h after application of a scratch to a layer of mMC with or without rAREG (**c**) Representative pictures of wound closure at 48 h after application of a scratch to a layer of murine tubulus cells (mTC) with or without rAREG. The remaining wound area is hatched and circled by the red line. (**d**) Quantification of wound closure at 48 h after application of a scratch to a layer of mTC with or without rAREG. (**e**) Representative pictures of wound closure at 8 h after application of a scratch to a layer of human glomerular endothelial cells (hGEnC) with or without rAREG. The remaining wound area is hatched and circled by the red line. (**f**) Quantification of wound closure at 8 h after application of a scratch to a layer of hGEnC with or without the addition of rAREG. Diamonds represent independent biological replicates and horizontal lines show mean values. Error bars show the standard error of the mean. **p < 0.01, ***p < 0.001.

The data presented above emphasize the critical role of Treg-derived AREG in protecting against renal damage and fibrosis in LN. To explore, whether these effects result from AREG’s direct influence on tissue repair, an in vitro scratch assay was conducted, using murine mesangial cells (mMC) and murine tubulus cells (mTC). Remarkably, both mMC (Fig. 7a, b) and mTC (Fig. 7c, d) displayed significantly improved wound healing in the presence of recombinant AREG, compared to the control condition. Extending this analysis to human glomerular endothelial cells (hGEnC), which are crucial for glomerular healing, similarly demonstrated accelerated wound closure with the addition of recombinant AREG after 8 h (Fig. 7e, f).

**Fig. 8:**
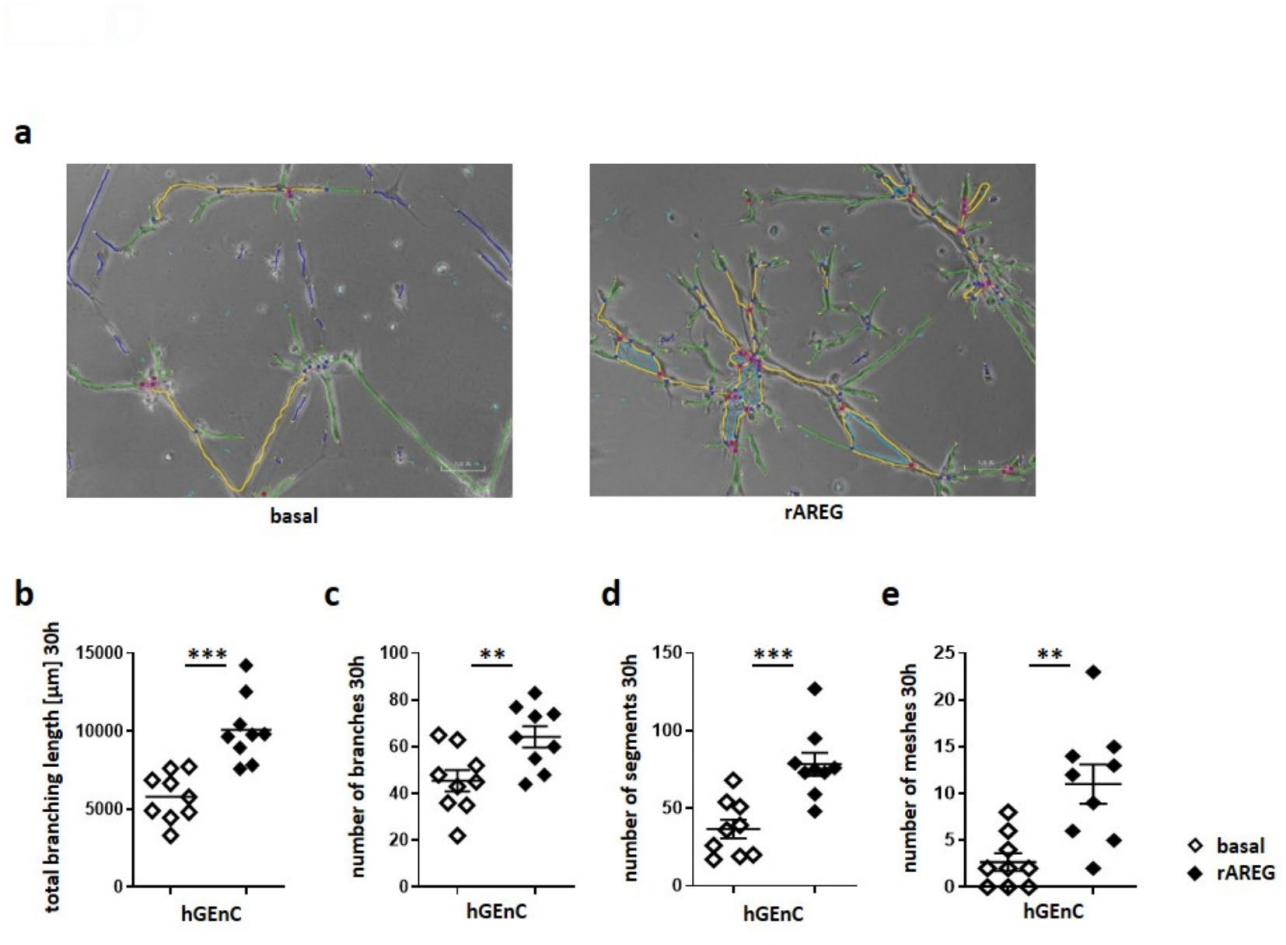
Recombinant AREG enhances angiogenesis. (**a**) Representative pictures of tube formation assays of hGEnC with or without the addition of recombinant AREG (rAREG) after 30 h. Branches are shown in green, segments are shown in yellow, and vascular meshes are shown in light blue. (**b**-**e**) Quantification of (**a**) total branching length, which is the sum of the length of branches and segments, (**c**) number of branches, (**d**) number of segments and (**e**) number of meshes. Diamonds represent individual cell culture preparations; horizontal lines show mean values. Error bars show the standard error of the mean. **p < 0.01, ***p < 0.001.

Finally, the impact of AREG on the growth of hGEnC was explored through a tube formation assay on matrigel, promoting 3D capillary-like structures. Quantitative analysis revealed, that recombinant AREG significantly enhanced vascular growth, as evidenced by increased total branching length, number of branches, number of vessel segments and number of meshes after 30 h (Fig. 8a-e).

## Discussion

Our previous investigation uncovered a potent protective role of AREG in the pristane-induced model of LN. Pan-knockout of AREG substantially exacerbated renal inflammation, highlighting its regulatory role. Notably, we found a direct downregulation of pathogenic Teff cell activation and cytokine production through AREG/EGFR signaling^10^. It remained unclear, though, whether other, non-immune mediated AREG functions might also contribute to the observed protection from LN. Furthermore, our study did not address the question, AREG secreted by which cellular source was responsible for the observed protective effects. We thus aimed to close these gaps using cell type-specific AREG knockout mice with a special focus on Tregs and M/M, since we had identified those leukocyte subtypes as the most prominent renal AREG producers in human LN^10^. We started off by generating FoxP3^cre^ x AREG^fl/fl^ mice with Treg selective AREG deficiency. PCR and FACS analysis confirmed the absence of AREG from Tregs while its expression was unchanged in other cell types. Next, we induced LN and studied outcomes at 12 and 15 months. Importantly, LN was exacerbated in mice lacking Treg-derived AREG, as evidenced by an increased number of abnormal glomeruli and increased glomerular area. Notably, this exacerbation also extended to interstitial damage at 15 months. Our data thus indicate a sustained protective role of Treg-derived AREG by mitigating kidney damage in pristane-induced LN. These protective effects were restricted to the context of LN since the glomeruli of naїve, age-matched FoxP3^cre^ x AREG^fl/fl^ mice were even slightly smaller than those of the wild-type controls. Importantly, the observed aggravation of renal injury also translated into an increase of renal fibrosis in mice lacking AREG in Tregs. This is in contrast to previous studies, which had demonstrated a pro-fibrotic role of AREG in renal disease^26,27,29^. These studies, however, assessed the effects of proximal tubule-derived AREG or the effects of pan-AREG blockade. Therefore, our findings once more underline the cell type-specific character of AREG´s functions with great implications for potential therapies. Next, we aimed to identify the mechanisms underlying the observed protective effects of Treg-derived AREG. Our previously published data studying LN in pan AREG KO mice had shown strong immunosuppressive effects of AREG on Teff^10^. Analyzing our FoxP3^cre^ x AREG^fl/fl^ mice, however, we did not observe relevant upregulation of renal or systemic T cell immune responses. On the contrary, IFNγ secretion by renal Teff was even slightly decreased in the absence of Treg derived AREG at the 12 month time point, despite aggravation of LN. The reason for this observation remains unclear. Reduction of IFNγ, however, was mild and was not seen systemically nor at 15 months after induction of LN, argueing against a general effect of Treg derived AREG on IFNγ responses. Furthermore, the absence of AREG from Tregs did not relevantly affect the M/M phenotype, which was previously shown to be skewed to a pro-inflammatory M1 phenotype by renal resident cell-derived AREG in another study by our group^23^. Along the same line, there were no changes in SLE characteristic humoral immunity, including serum anti-dsDNA antibodies as well as renal complement and immunoglobulin deposition in mice with AREG-deficient Tregs. Continuing our investigation, we next examined whether the observed functions of AREG are exclusive to Tregs, or if AREG from other sources elicits similar effects. In order to assess the impact of macrophage-secreted AREG in the pristane model of LN, LysM^cre^ x AREG^fl/fl^ mice were generated to eliminate AREG production in myeloid cells. However, in contrast to mice lacking Treg-derived AREG, mice deficient in myeloid cell-derived AREG did not display aggravation of renal damage or fibrosis at 12 months of pristane-induced LN. Furthermore, examination of the immunological phenotype revealed no notable variation of Teff cytokine production. In line with our previously published data^23^, however, we found a shift towards M2 polarization in macrophages from mice lacking myeloid cell-derived AREG. Since lack of myeloid cell-derived AREG did not alter the course of pristane-induced LN, the functional relevance of this observation seems to be negligible in this model. Overall it is interesting to note, that Treg but not M/M derived AREG relevantly influences the course of LN. This again highlights the notion, that AREG from different cellular sources has different functions. The reasons for this fact currently remain largely unclear but might include differences in spatial localization of AREG secreting cells, co-secretion of further mediators modulating AREG´s function, as well as cell type specific posttranslational modifications of AREG protein.

Taken together, our findings suggest, that specifically Treg-derived AREG mediates reno-protective effects in LN, without relevant alterations of SLE characteristic immune responses. In search of underlying mechanisms, we next studied apoptosis, since AREG is a pro-survival factor for many different cell types. Indeed, we found increased apoptosis of resident renal cells in the glomeruli and interstitium of nephritic mice lacking AREG secretion from Tregs. Conversely, Treg derived AREG significantly enhanced tubular regeneration. These protective effects of Treg-derived AREG in LN prompted further investigation into the responsible mechanisms, particularly focusing on AREG´s direct roles in tissue repair, which were suggested by previous studies^15,30^. Utilizing an in vitro scratch assay, we found improved wound healing of various renal cell types, including murine mesangial cells (mMC), tubular epithelial cells (mTC), and human glomerular endothelial cells (hGEnC), upon treatment with recombinant AREG. This finding indicates a direct influence of AREG on growth and regeneration of mesenchymal, epithelial, and endothelial cells. Furthermore, these in vitro data align with previous in vivo findings, demonstrating that Treg-derived AREG can enhance lung alveolar mesenchymal cell growth during influenza virus infection^31^. Further investigation, using a tube formation assay with hGEnCs revealed, that recombinant AREG also significantly facilitated angiogenesis. These results corroborate previous observations and highlight AREG’s pro-angiogenic properties. Interestingly, while previous studies reported EGFR-mediated vascular repair via pericytes^13,32^, our data indicate, that AREG can directly act on vascular endothelial cells without any intermediaries. This notion is consistent with recent findings in mouse aortic and human umbilical vein endothelial cells^33^. In conclusion, our study demonstrates a long-term reno-protective effect of Treg- but not M/M derived AREG in LN. Mechanistically, we found that AREG protects from apoptosis and enhances renal tissue regeneration as well as vascular repair to prevent detrimental fibrosis. These findings further underscore the great potential of Treg directed therapies and the AREG/EGFR axis in LN.

## Methods

### Animals

Mice carrying a floxed AREG gene (Aregtm2a(EUCOMM)Hmgu) were kindly provided by Dietmar M. Zaiss, Regensburg, Germany, and were initially generated by the MRC Harwell Institute, UK^13,15^. The B6(Cg)-Foxp3tm4(YFP/icre)Ayr/J mice expressing cre recombinase under the control of the Foxp3 promotor were graciously provided by Alexander Y. Rudensky of Memorial Sloan-Kettering Cancer Center, New York^25^. Mice expressing LysM-driven cre recombinase (B6.129P2-Lyz2tm1(cre)Ifo/J) were kindly provided by Gisa Tiegs, Hamburg, Germany and initially derived from the Jackson Laboratory, Bar Harbour, ME, USA. Both cre-containing strains were cross-bred with AREG floxed mice to achieve Treg- or myeloid cell-specific deletion of AREG. Genomic DNA from tail snips of all mice was PCR analyzed as outlined below to confirm the correct genotypes. All mice were maintained on a C57BL/6J background and were bred in-house under specific pathogen-free conditions.

### Animal experiments

For the lupus nephritis model, 8–12-week-old female mice of the specified genotypes were intraperitoneally injected once with 500 µl of pristane oil (2,6,10,14-tetramethylpentadecane). Analysis of pristane-induced peritonitis took place after 7–14 days, while assessment of chronic lupus nephritis occurred at 12 and 15 months post-injection. Mice were humanely culled at the indicated time points using isoflurane narcosis and cervical dislocation. All animal experiments were conducted by national and institutional animal welfare and ethics guidelines and received approval from the local ethics committee at the ´Behörde für Justiz und Verbraucherschutz Hamburg, Abt. Lebensmittelsicherheit und Veterinärwesen´ (approval codes N081/2020, N21/005). The study is reported in accordance with ARRIVE guidelines.

### Isolation of leukocytes from different tissues

The spleens were harvested and passed through a 70-µm nylon cell strainer immersed in Hanks’ Balanced Salt Solution (HBSS). To remove red blood cells, ammonium chloride was employed for lysis. Subsequently, the cells underwent a washing process and were filtered through a 40-µm nylon cell strainer. The number of cells was quantified using an automated cell counter. For the extraction of leukocytes from the kidneys, the tissue was finely chopped and exposed to a digestion solution containing DNAse and collagenase at 37 °C for 45 min. Subsequently, a gentleMACS™ dissociator was employed to dissociate the tissue. Subsequent purification was achieved by subjecting the sample to a 37% Percoll gradient. Cells from the peritoneum were harvested by injecting a total of 5 ml PBS containing 3% FBS. The peritoneum was then washed by pressure movements and the 5 ml lavage was subsequently completely removed again after a small incision in the peritoneum. After spinning the lavage for 5 min at 350 g the cells were resuspended in HBSS and passed through a 40-µm nylon cell strainer.

### Flow cytometry

Cells were surface-stained for 20 min at 4 °C with fluorochrome-labeled antibodies against CD45 (30-F11), CD4 (RM4-5), CD44 (IM7), CD69 (H1.2F3), CD11b (M1/70), CD11c (N418/HL3) (all BD Biosciences, Franklin Lakes, NJ), CD3 (500A2), CD8 (53 − 6.7), CD19 (6D5), CD62L (MEL-14), γδ T cell receptor (eBio GL3), Ly6C (AL-21), Ly6G (1A8) (all BioLegend, San Diego, CA). For intracellular staining, cells were fixed in 3.65% paraformaldehyde (PFA) for 20 min at room temperature (RT) and subsequently permeabilized with 0.1% Nonidet P-40 (ThermoFisher Scientific, Waltham, MA) for 4 min at RT. For intracellular/intranuclear staining, fluorochrome-labeled antibodies against IL17A (TC11-18H10.1), IFNγ (XMG1.2), (eBioscience, San Diego, CA), TNFα (MP6-XT22), Ki-67 (B56) (BD Biosciences, Heidelberg, Germany), IL-13 (eBio13A) and FoxP3 (FJK16s) (Invitrogen) were used. For staining of intracellular AREG, we used a biotinylated anti-AREG antibody (BAF989; R&D Systems, Minneapolis, MN) and a fluorochrome-labeled streptavidin afterward (BioLegend, San Diego, CA). For the ex vivo cytokine stimulation of T cells, the cells were activated with phorbol-12-myristat-13-acetat (PMA) (50 ng/mL; Sigma-Aldrich), Ionomycin (1 µg/mL; Calbiochem-Merck) and Brefeldin A (10 µg/mL; Sigma-Aldrich, St. Louis, MO) for 2.5 h at 37 °C. For the ex vivo cytokine stimulation of macrophages, the cells were activated with Lipopolysaccharide (LPS) (1 µg/mL; Sigma-Aldrich, St. Louis, MO), Adenosintriphosphat (ATP) (10 µM; Cytoskeleton) and Brefeldin A (10 µg/mL; Sigma-Aldrich, St. Louis, MO) for 2.5 h at 37 °C.

For Annexin V staining, surface stained cells were suspended in BioLegend Annexin V Buffer with a dilution of 1:50 of Annexin V Antibody (BioLegend, San Diego, CA). The cells were then washed in BioLegend Annexin V Buffer and immediately analyzed by Flow cytometry. LIVE/DEAD staining (Invitrogen, Carlsbad, CA or ThermoFisher Scientific, Waltham, MA) was used to exclude dead cells in all analyses. Experiments were performed on a BD FACSymphony A3 (BD Biosciences, Heidelberg, Germany).

### Cell sorting

In experiments involving primary mouse B and T cells, the isolation process was performed by FACSorting. Single-cell suspensions were prepared from spleens, following the previously mentioned procedure. These cells were then stained with antibodies targeting B cells (CD19) and T cells (CD3 and CD4) to specifically isolate distinct leukocyte populations. Fluorescence emitted by the FoxP3^cre/yfp^ construct was employed to identify and sort regulatory T cells. The sorting was performed in cooperation with the institutional FACSorting Core facility.

### Genomic DNA extraction and PCR analysis procedures

Genomic DNA from mousetails was extracted using a proteinase K-based protocol. A piece of the tail was suspended in 100 µl lysis buffer, containing proteinase K at a 1:50 dilution. This mixture was incubated at 55 °C for four hours in a thermoblock. The proteinase K reaction was terminated by heating the mixture to 85 °C for 45 min. For analysis of genomic DNA from sorted cells, these were resuspended in 200 µl lysis buffer containing 1:100 Proteinase K. This suspension was placed in a 55 °C warm thermoblock for 1 h and subsequently centrifuged for 10 min at 12,000 rpm at room temperature (RT). The supernatant was collected and transferred into a tube containing 200 µl isopropanol. The tube was inverted until DNA precipitation occurred and then centrifuged for 10 s at 4,000 rpm at RT. The DNA underwent two washes with 500 µl 70% EtOH, and after each wash, it was centrifuged for 10 s at 4,000 rpm at RT. In the final step, the DNA was centrifuged for 10 min at 4,000 rpm at RT. The supernatant was discarded, and the DNA was air-dried at RT for approximately 15 min. The dried DNA was then resuspended in 50 µl H_2_O and incubated in a 55 °C thermoblock for 1 h. For PCR analysis, DNA concentration was assessed using a nanodrop, and equal amounts for each sample were utilized. PCR products were examined on a 2% TBE-agarose-ethidium-bromide gel under UV light. Primer sequences are available upon request.

### ELISA analyses of serum antibodies

Retrobulbar blood was gathered in ethylenediaminetetraacetic acid (EDTA) coated tubes, followed by centrifugation at 3500 rpm for 15 min. Subsequently, circulating anti-ds-DNA antibodies from serum were measured by ELISA at the indicated dilutions after coating microtiter-plates with Poly-L-Lysine (Sigma-Aldrich, St. Louis, MO) and calf thymus DNA (Worthington, Lakewood, NJ). For analysis of total non-antigen-specific immunoglobulins, ELISA plates were precoated with anti-mouse IgG antibodies (Jackson Immuno Research, West Grove, PA). As a secondary antibody, anti-total-IgG (Southern Biotech, Birmingham, AL) was used. Additionally, ELISA for anti-U1-snRNP in the serum was conducted at specified dilutions after coating the microtiter plates with snRNP.

### Morphological studies

Glomerular abnormalities were determined in a minimum of 50 glomeruli per mouse in 1.5-µm-thick PAS-stained kidney sections in a blinded manner as published^34^. These included glomerular hypercellularity, crescent formation, fibrinoid necrosis, segmental proliferation, hyalinosis, and capillary wall thickening. The glomerular area was measured in a minimum of 50 glomeruli on high-power images (magnification x400) with AxioVision Rel. 4.8 LE^35^. Semiquantitative analysis of tubulointerstitial damage using a score from 0 to 4 was performed using 20 randomly selected cortical areas (x200) as published previously^36^.

### Immunohistochemistry

Paraffin-embedded sections were stained with antibodies directed against cleaved caspase-3 (Asp175, 5A1E; Cell Signaling Technology, Danvers, MA), C3 (C3c, A 0062; Dako, Hamburg, Germany), Ki67 (D3B5, Cell Signaling, Danvers, MA) or mouse IgG (polyclonal; Dianova, Hamburg, Germany) and developed with a polymer-based secondary antibody-alkaline phosphatase kit (POLAP; Zytomed, Berlin, Germany). Cells positive for caspase-3 or mouse IgG were counted in a blinded fashion in 50 glomerular cross-sections (gcs) and 20 tubulointerstitial high power fields (hpf, magnification x 400) per kidney section. For glomerular C3 deposition, 50 glomeruli were blindly evaluated in a semiquantitative manner. Each glomerulus was scored from 0 (no staining) to 4 (maximal staining intensity).

### Fibrosis staining

To visualize fibrotic tissue, kidney sections measuring 3 μm in thickness and embedded in paraffin were subjected to staining with Sirius Red for one hour. Following this procedure, the sections were subjected to Fast Green staining for an additional hour. Subsequently, an ascending alcohol series was applied, and the sections were cover-slipped with Eukit. Images of the slides were captured at random and analyzed using ImageJ software to quantify the red staining.

### Culture of human glomerular endothelial cells

An immortalized human glomerular endothelial cell line (hGEnC) was provided by Simon C. Satchell, Bristol Medical School, Bristol, United Kingdom. Primary hGEnCs were immortalized by transfection with SV-40 virus^37^. hGEnC were cultured in EGM™-2 Endothelial Cell Growth Medium-2 by Lonza in 5% CO_2_ at 33 °C. The hGEnC were trypsinated, counted, and seeded in a 6-well plate at a density of 3 × 10^5^ cells/well for cell culture experiments.

### Culture of murine mesangial and proximal tubulus cells

Mouse kidney proximal tubule cells (mTC) originate from a cell line obtained through microdissection from the mouse proximal tubule and were subsequently immortalized via transformation with the SV-40 virus^38^. Mouse mesangium cells (mMC) were initially derived from mouse kidneys using differential sieving and digestion techniques and were also immortalized through SV virus transformation^39^. The cells were cultured in DMEM medium supplemented with 10% FCS, 100 U/ml penicillin, and 100 mg/ml streptomycin at 37 °C, and 5% CO₂. Confluent mTC and mMC were detached for cell culture experiments using trypsin and then seeded in a 6-well plate at a dilution of 1:10 for mMC and 1:15 for mTC. Before stimulation, the cells were incubated in serum-free DMEM for 24 h.

### Scratch assay

Cells were seeded in 6-well plates, following the above described procedure, and cultured at 5% CO₂ and 37 °C for an additional 1–2 days until reaching confluence. Subsequently, the respective wells were longitudinally scratched using a 100 µl pipette tip. After removing the medium, fresh medium was applied with or without the addition of 50 ng/ml recombinant human or murine AREG. Images capturing the scratch were taken at time 0 h using an inverted microscope. The cells were then cultured further at 5% CO₂ and 37 °C for an additional 6 h in the case of hGEnC and 48 h in the case of mMC and mTC. Following this incubation period, additional images were captured using an inverted microscope. Wound healing was assessed using a wound healing analysis plug-in for ImageJ^40^.

### Endothelial cell tube formation assay

hGEnCs were subconfluently seeded and provided with extracellular matrix support. A GeltrexTM matrix-coated 24-well plate was prepared and allowed to cure at 37 °C for 30 min. Subsequently, 7.5 × 10^4^ hGEnC were mixed with medium, with or without 50 ng/ml recombinant human AREG. A 300 µl portion of this mixture was added to each well and cultured at 5% CO₂ and 37 °C for 30 h. Images were captured using an inverted microscope, and tube formation was quantified utilizing the Angiogenesis Analyzer PlugIn in ImageJ^41^.

### Statistical analyses

Results are presented as mean ± standard error of the mean (SEM). To compare two groups, the Student’s t-test was used. Statistical significance was determined by a P-value < 0.05.

## Electronic supplementary material

Below is the link to the electronic supplementary material.

Supplementary Material 1

## Data Availability

All data supporting the findings of this study are available within the paper and its Supplementary Information.
