# Supplementary material for: Treg derived Amphiregulin protects from murine lupus nephritis via tissue reparative effects
Source: Sci Rep. 2025 Mar 5;15:7776. doi: 10.1038/s41598-025-91636-2 (PMC11882795; doi:10.1038/s41598-025-91636-2)
Supplement: Supplementary file 1 — Supplementary Material 1 [file 41598_2025_91636_MOESM1_ESM.pdf]

Supplementary Figure S1

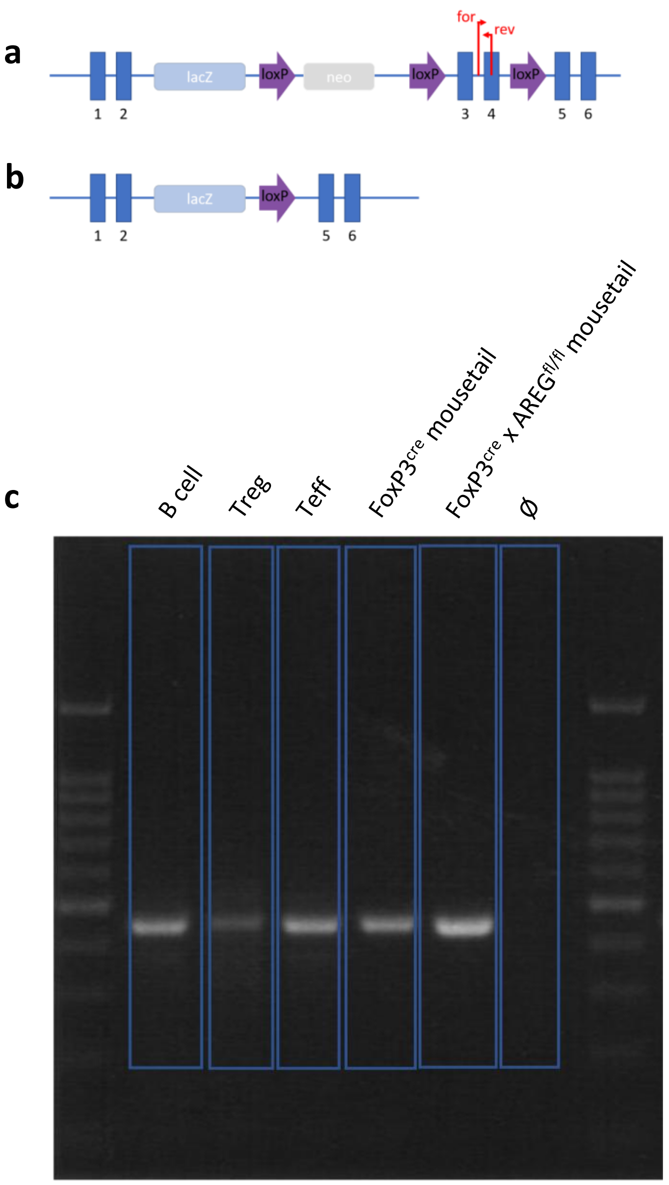

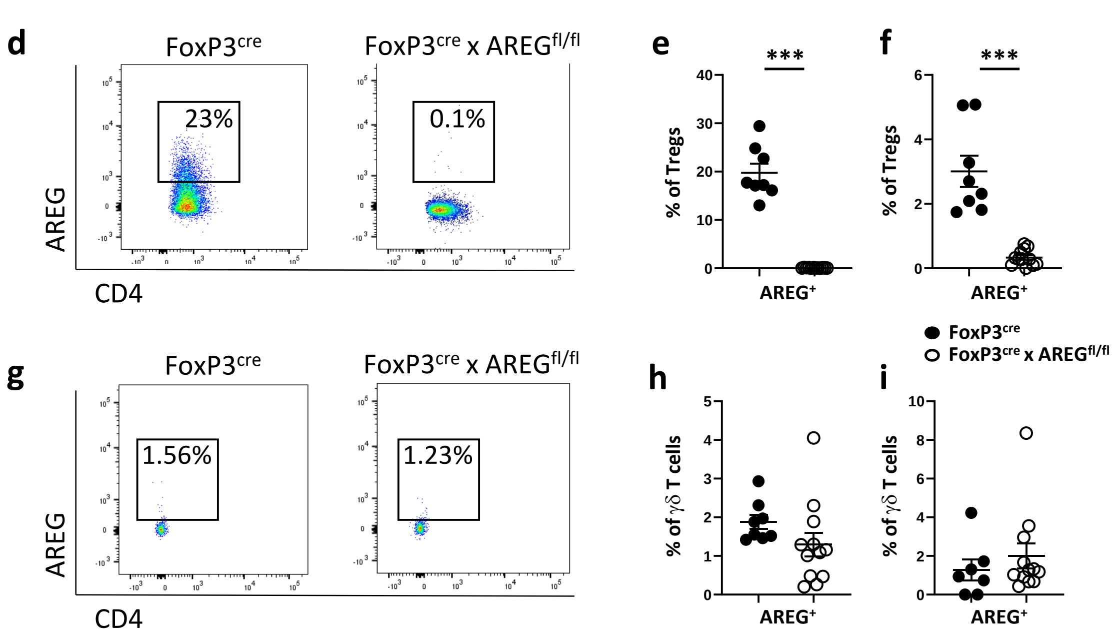

<u>Supplementary Figure S1. Lack of AREG in FoxP3^{cre} x AREG$^{fl/fl}$ mice is limited to Tregs</u>

(a) The genomic structure of the Areg$^{tm2a(EUCOMM)Hmgu}$ mouse with a floxed AREG gene adapted from the IMPC website (www.mousephenotype.org). Exons are numbered 1-6 and indicated by blue bars. LacZ and neomycin genes (neo) are shown as light blue and grey rectangles respectively. The three floxed (loxP) sites are shown by dark purple arrows. The position of the primers used in our analyses is marked by red arrows with the forward (for) primer binding within the intron between exons 3 and 4 and the reverse (rev) primer binding within exon 4. (b) The expected genomic structure after cre-mediated recombination with excision of the critical exons 3 and 4. (c) PCR analysis of the AREG gene from genomic DNA of highly purified splenic B cells, Tregs, and T effector cells (Teff) of FoxP3^{cre} x AREG$^{fl/fl}$ mice, and of tail tissue from wild type mice and FoxP3^{cre} x AREG$^{fl/fl}$ mice. (d) Representative FACS plot of AREG^{+} Tregs in the spleen of mice at 15 months of pristane-induced LN. (e-f) Quantification of AREG^{+} Tregs in (e) the spleen and (f) the kidney of mice with the indicated genotype at 15 months of pristane induced LN. (g) Representative FACS plot of AREG expression by γδTCR^{+} T cells in the spleen of control mice. (h-i) AREG^{+} γδ T cells in (h) spleen and (i) kidneys of mice with the indicated genotype at 15 months of pristane-induced LN. Circles show individual animals, horizontal lines show mean values. Error bars show the standard error of the mean. ***$p<0.001$

Supplementary Figure S2

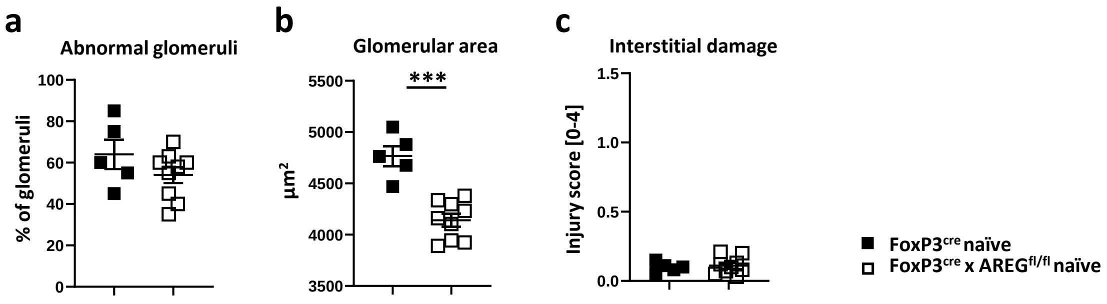

<u>Supplementary Figure S2. Analysis of the glomerular area in aged naïve mice</u>

Naïve mice were analyzed at 14.5 months of age, matching the age of mice at 12 months after induction of pristane-induced LN. Quantification of a) abnormal glomeruli, b) the glomerular area and c) interstitial damage of age matched naïve wild type and FoxP3^{cre} x AREG$^{fl/fl}$ mice. Rectangles show individual animals, horizontal lines show mean values. Error bars show the standard error of the mean. ***$p<0.001$

Supplementary Figure S3

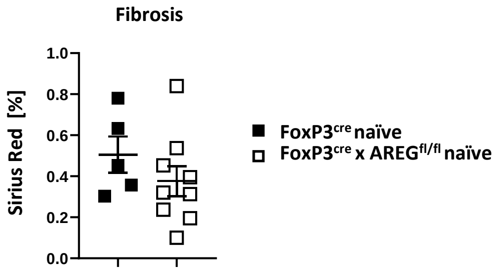

Supplementary Figure S3. Quantification of fibrosis in aged naïve mice

Naïve mice were analysed at 15 months of age, matching the age of mice at 12 months after induction of pristane-induced LN. Quantification of the fibrotic area in kidneys of age matched naïve wild type and $FoxP3^{cre}$ x $AREG^{fl/fl}$ mice. Rectangles show individual animals, horizontal lines show mean values.

Supplementary Figure S4

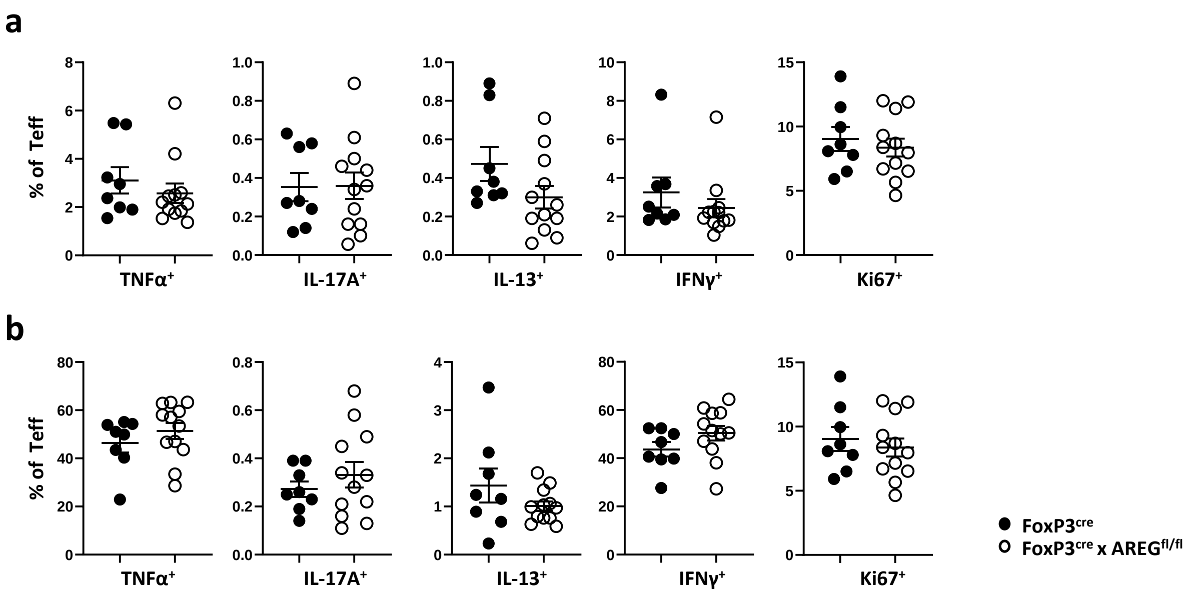

<u>Supplementary Figure S4. Cytokine production and proliferation by renal and splenic Teff</u>

(a) Cytokine production and proliferation (Ki67) of Teffs in kidneys from mice of the indicated strains at 15 months of pristane-induced LN. (b) Cytokine production and proliferation of Teffs from spleens of indicated strains at 15 months of pristane-induced LN. Circles show individual animals, horizontal lines show mean values. Error bars show the standard error of the mean.

Supplementary Figure S5

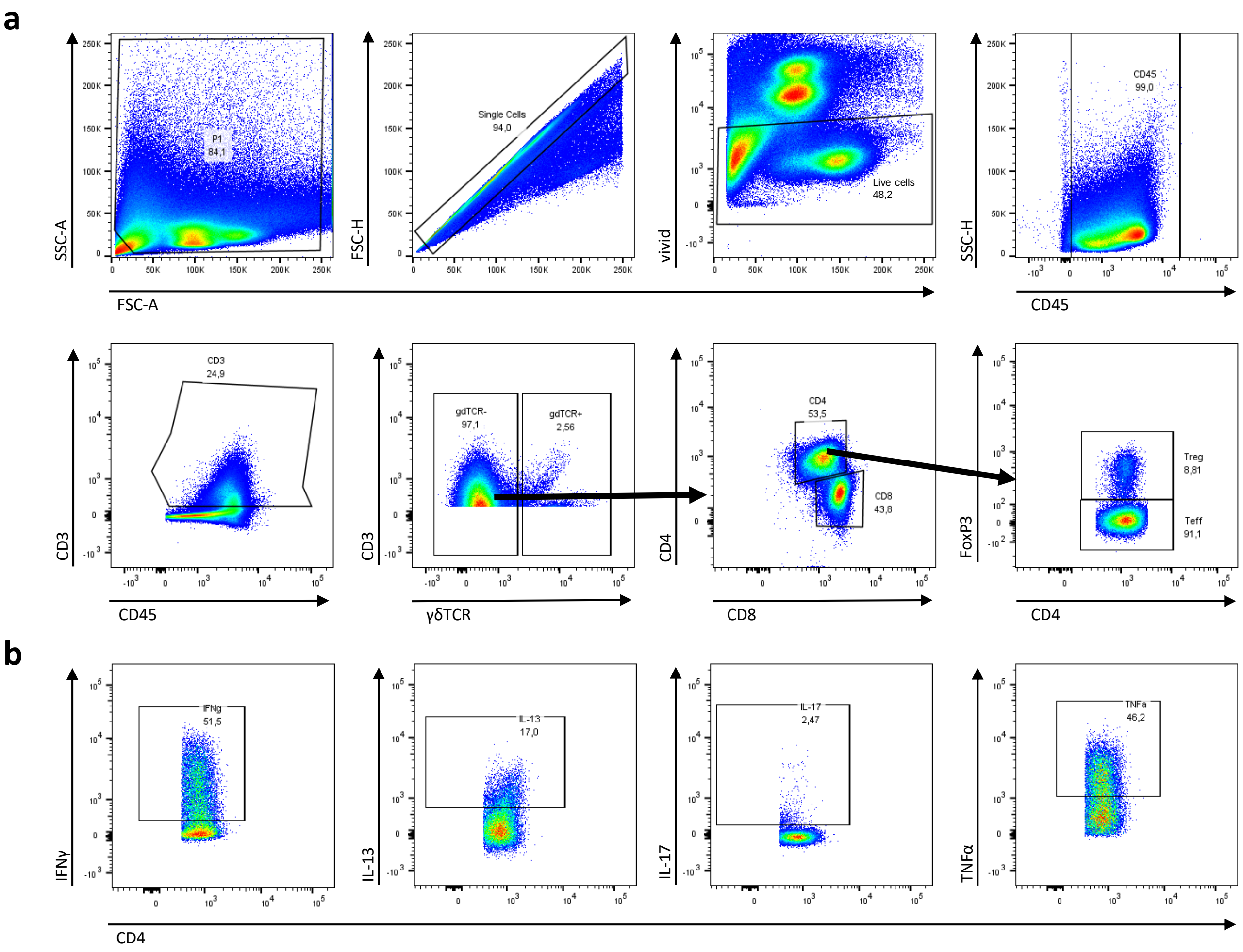

Supplementary Figure S5. Gating strategy of T cell subtypes

(a) Single cell suspensions from kidneys of nephritic mice were analyzed by flow cytometry after *ex vivo* stimulation with PMA/ionomycin. After exclusion of doublets and dead cells, leukocytes were gated as indicated. (b) Production by the indicated cytokines by Teff was assessed. Treg: Regulatory T cell, Teff: T helper effector cell

Supplementary Figure S6

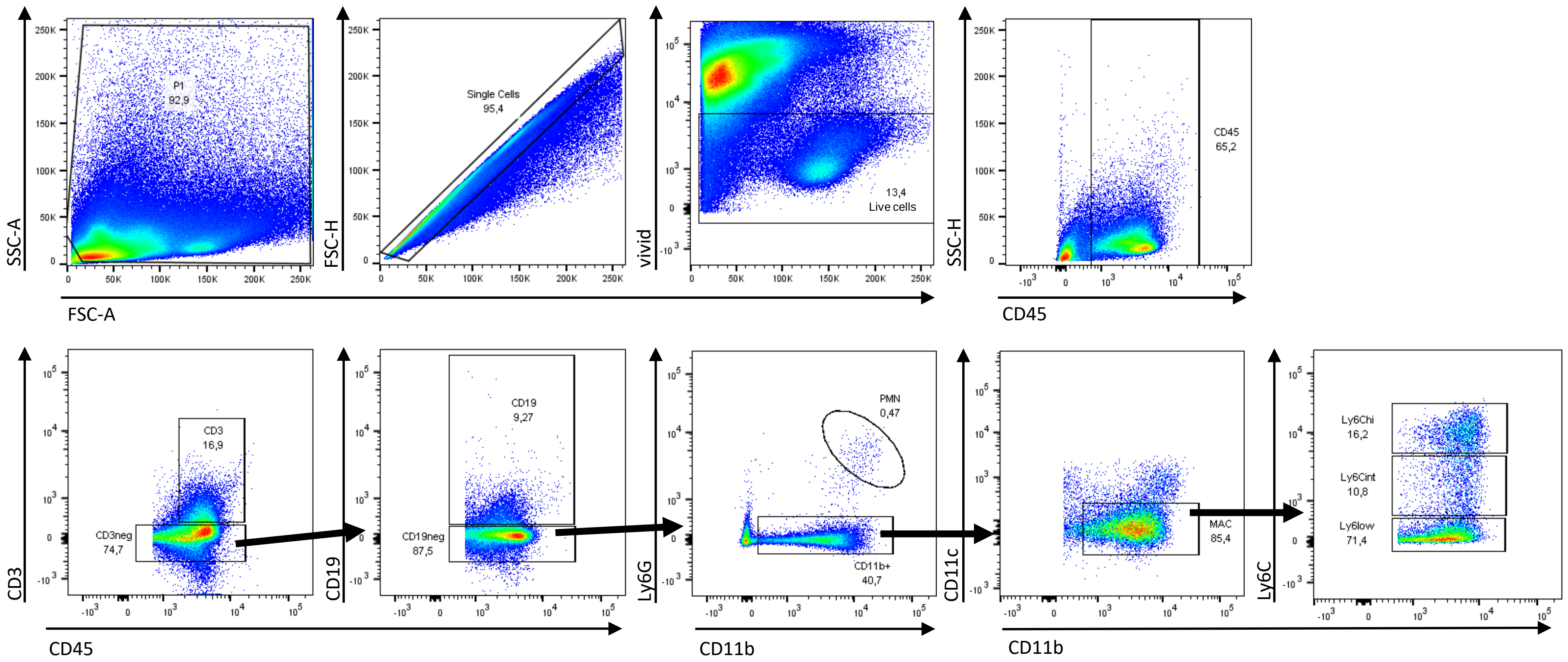

Supplementary Figure S6. Gating strategy of myeloid cells

Single cell suspensions from kidneys of nephritic mice were analyzed by flow cytometry. After exclusion of doublets, dead cells, $CD3^+$ T cells, $CD19^+$ B cells, $Ly6G^+$ neutrophils (PMN) and $CD11c^+$ dendritic cells, $CD11b^+$ monocytes/macrophages were gated according to the Ly6C status.

Supplementary Figure S7

a

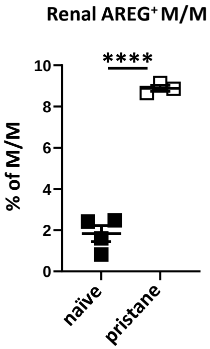

<u>Supplementary Figure S7. AREG production by renal M/M in LN</u>

(a) Naïve mice were analysed at 14.5 months of age, matching the age of mice at 12 months after induction of pristane-induced LN. FACS analyses shows strong expansion of renal infiltrating AREG producing M/M at 12 months of pristane induced LN. Rectangles show individual animals, horizontal lines show mean values. Error bars show the standard error of the mean. ****p<0.0001

Supplementary Figure S8

a

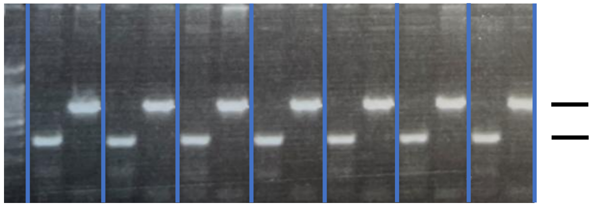

b

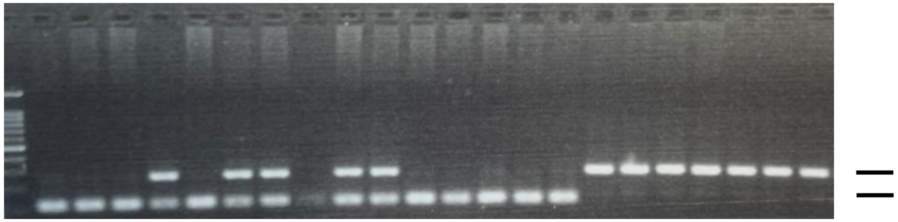

<u>Supplementary Figure S8. Genotyping of LysM^{cre} x AREG$^{fl/fl}$ mice</u>

(a) Examples showing PCR analysis of the LysM gene, using genomic DNA extracted from murine tail snips. Each analyzed mouse is heterozygous for the LysM^{Cre} construct (700bp) and the wild type allele (350bp). (b) Examples showing PCR analysis of the AREG gene, using genomic DNA extracted from murine tail snips. Mice are either homozygous for the AREG^{flox} construct (one band at 120bp), homozygous wild types (one band at 263bp) or heterozygous (one band at 120bp and one band at 263bp). Only mice heterozygous for LysM^{Cre} and either homozygous for AREG^{flox} or the AREG wild type allele were used for our studies. bp: base pair

Supplementary Figure S9

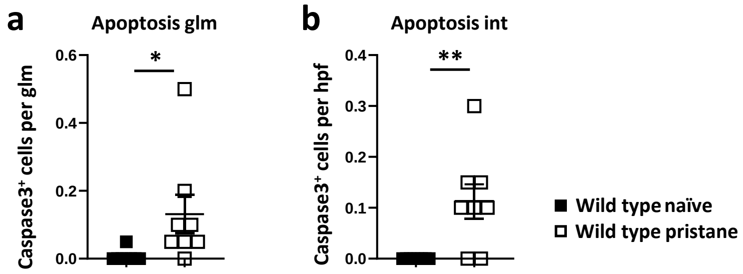

<u>Supplementary Figure S9. Analysis of apoptosis induced by pristane</u>

Naïve mice were analysed at 14.5 months of age, matching the age of mice at 12 months after induction of pristane-induced LN. Quantification of a) glomerular and b) interstitial caspase3^{+} apoptotic cells in kidneys of naïve versus pristane treated mice. Rectangles show individual animals, horizontal lines show mean values. Error bars show the standard error of the mean. *p<0.05, **p<0.01
